# Honey, they shrunk the ATP

**DOI:** 10.1073/pnas.2410446121

**Published:** 2024-06-27

**Authors:** David Eisner, Elizabeth Murphy

**Affiliations:** ^a^Division of Cardiovascular Sciences, The University of Manchester, Manchester M13 9NT, United Kingdom; ^b^Cardiac Physiology Laboratory, National Heart, Lung, and Blood Institute, NIH, Bethesda, MD 20892

As we learned at school, adenosine triphosphate (ATP) is the energy currency of all cells in the body. In the heart, it is used to fuel the interactions between action and myosin which produce contraction and the pumping of blood, as well as to establish the transmembrane gradients of calcium that activate this process. In PNAS, Rhana et al. (1) now report that the concentration of ATP ([ATP]) is at least 10 times lower than previously thought, a finding with profound implications.

When used to power cellular functions, ATP is hydrolyzed to adenosine diphosphate (ADP) and inorganic phosphate (P_i_). It has been estimated that the myocardium contains only enough ATP to power contraction for about 20 beats (2). ATP must therefore be continually regenerated, largely by oxidative phosphorylation in mitochondria. The free energy available from the hydrolysis of ATP depends on the ratio of concentrations of ATP to ADP and P_i_. When metabolism is compromised, this ratio falls, and during ischemia, the [ATP] falls to low levels (3, 4). As a consequence, the actomyosin ATPase which powers contraction will be slowed, and the activities of ATP-dependent ion pumps will decrease with a resulting decrease in the Ca^2+^ content of the sarcoplasmic reticulum (SR) and increases in cytoplasmic Ca^2+^ and Na^+^ concentrations. Cardiac myocytes also express an ATP-sensitive potassium channel (K_ATP_). As [ATP] decreases, these channels will open, decreasing the duration of the action potential (5). Therefore, both contractility and excitability are intimately linked to [ATP].

Given the above, it is important to know what the concentration of ATP is. Initial studies used rapid freezing of the heart and then measured [ATP] with chemical methods to obtain estimates of about 8 mM (see ref. 6 for references). This approach is destructive, making it impossible to obtain serial measurements and study the time course of change of [ATP]. One study froze hearts either in diastole or systole and found small reductions of [ATP] in systole (7). Much subsequent work has used a noninvasive technique, ^31^P NMR, and obtained similar values for [ATP] (6). While NMR allows repetitive measurements, its lack of sensitivity means that it takes of the order of a minute to obtain an accurate measurement of [ATP], with the problem being most severe for small hearts such as those from mouse. It is, however, possible to “gate” the data acquisition to the heartbeat and, by averaging data from many beats, obtain a time course of at least a few samples per heartbeat. With this approach, it has been shown that [ATP] decreases on each beat (8–10). Another ^31^P NMR study, however, found no change of [ATP] during the cardiac cycle (11).

The amount of tissue required for the above methods precludes their use at the single-cell level. Another approach used luciferase injected into a single ventricular myocyte in the presence of luciferin to measure [ATP] (12), but technical difficulties have limited its subsequent use. A major advance in this area has been made by Rhana et al. (1) using a novel fluorescent sensor (13) to measure [ATP]. This allowed selective measurements of cytoplasmic [ATP] from a single cell with rapid time resolution. This approach immediately produced a very surprising result: in diastole [ATP] was about 500 µM, at least an order of magnitude lower than the values reported above. A common problem when using indicators inside cells is that their properties may be different in the cytoplasmic environment compared to when studied in simple salt solutions (14). Is it therefore possible that the apparent low [ATP] results from a lower affinity of the indicator in the cell? Rhana et al. excluded this possibility with a careful in situ calibration dialyzing different concentrations of ATP into the cell. Assuming that the cytoplasmic [ATP] equilibrates with that in dialyzing pipette, and there is no hydrolysis of the added ATP, the affinity was as expected.

What is the explanation of the cytoplasmic [ATP] being so much lower than in previous studies? One possibility, suggested by the authors, is that most of the ATP measured by previous work is not free in the cytoplasm but either bound to proteins or sequestered within intracellular stores, with only a small cytoplasmic component sensed by the indicator. Any binding sites on proteins would have to be present at a much greater concentration than those at which ATP is hydrolyzed by the SR Ca-ATPase or myosin (typically found at concentrations of the order of 100 µM). Furthermore, the similar values of [ATP] measured in chemical studies (which report all ATP) and ^31^P NMR which would not be expected to detect bound ATP suggests that the bound fraction is rather small (15). One potential intracellular store for ATP is provided by mitochondria which occupy about one-third of the ventricular cell volume. Taking the average cell [ATP], measured by NMR as 7.5 mM, then the ATP concentration in the mitochondria would need to be 21.7 mM to fit with a cytoplasmic concentration of 0.5 mM. One issue is, however, troubling. Inhibiting mitochondrial oxidative phosphorylation with cyanide has no measurable effect on total cellular [ATP] as measured with ^31^P NMR (16). Presumably, under these conditions, mitochondrial and cytoplasmic [ATP] become more equal, with mitochondrial and cytoplasmic [ATP], respectively, decreasing and increasing. One would therefore predict that inhibiting oxidative phosphorylation would increase cytoplasmic [ATP]. This seems improbable, but future studies are required to measure the effects of such inhibition on cytoplasmic and (with an appropriate indicator) mitochondrial [ATP].

An important functional consequence of the low [ATP] is that it may be low enough to activate a fraction of the K_ATP_ channels, more so than if [ATP] was several mM. Consistent with this, Rhana et al. find that the application of the K_ATP_ inhibitor, glibenclamide, results in afterdepolarizations which are known to be arrhythmogenic, suggesting that the low [ATP] may protect against arrhythmias.

In PNAS, Rhana et al. now report that the concentration of ATP ([ATP]) is at least 10 times lower than previously thought, a finding with profound implications.

Rhana et al. find large changes (~50%) of [ATP] during contraction. These peak in about 30 to 40 ms and decay within 100 ms. However, the direction of these [ATP] transients was not usually what would be expected from the ^31^P NMR work. In about half the cells (Mode 1), [ATP] increased and decreased in only a minority (16%) (Mode 2). In the remainder of the cells, some regions showed an increase and others a decrease. The increases and decreases of [ATP] were associated with, respectively, increases and decreases of mitochondrial calcium concentration, as measured with the indictor dh-Rhod2. Downregulation of mitofusin 2, to decrease the linkage between SR and mitochondria, decreased the amplitude of the increases of both mitochondrial Ca^2+^ and cytoplasmic [ATP]. The authors conclude that in Mode 1, Ca^2+^ release from the SR increased mitochondrial Ca, perhaps because those mitochondria are very close to the SR, leading to an increased activity of mitochondrial tricarboxylic acid cycle activity and hence an increase of ATP production. The idea is that it is these mitochondria which are responsible for increasing ATP supply to meet demand. In Mode 2, the mitochondria are suggested to be too far from the SR for this mechanism to occur and the metabolic demands of contraction and Ca^2+^ cycling decrease [ATP]. Future work should examine the underlying mechanism. It is also worth noting that the energy consumption of isolated myocytes is less than when the heart is performing external work. Does this mean that in the intact heart, all myocytes will show a decrease of [ATP] during systole?

In addition to being an energy source, ATP interacts with both magnesium and calcium ions with most of the cytoplasmic Mg^2+^ bound to ATP. ATP binds Ca^2+^ with a comparatively low affinity under cellular conditions (apparent K_d_ ~ 2.5 mM). Although it will therefore bind little Ca^2+^ at cytoplasmic Ca^2+^ concentrations (0.1 to 1 µM), it may be a more important Ca^2+^ binder at the higher Ca^2+^ concentrations near the SR Ca^2+^ release sites (see ref. 17 for review). The concentration of Ca-ATP is higher than that of Ca^2+^ and diffusion of Ca-ATP will speed up the rate of diffusion of Ca^2+^ from the SR release sites to the myofilaments, thereby accelerating activation of contraction. This has been demonstrated in skeletal muscle (18) but would also be expected to be important in the heart. The low value of [ATP] reported here (1) will virtually abolish the contribution to diffusion of Ca^2+^. In this context, it will be interesting to find whether cytoplasmic [ATP] is as low in skeletal muscle as now measured in cardiac myocytes.

Finally, alterations of high-energy phosphorous metabolites have been implicated in heart failure. As reviewed previously (19), [ATP] decreases in the later stages of heart failure by about 40%, corresponding to a level of 4 mM. The K_m_ of SERCA for ATP is at most a few hundred micromolar (20) so this decrease of [ATP] would not be expected to have much effect on Ca^2+^ pumping. If, however, the normal [ATP] is as low as reported by Rhana et al., then the effects of the same fractional decrease of [ATP] may be more significant. Likewise, the lower [ATP] may well be significant for other ATPases, in particular the actomyosin ATPase (21).

In summary, the results of Rhana et al. have major implications for our understanding of metabolism, not only in the heart but potentially also in other organs. There is much for others to follow up.
